# QuickStats

**Published:** 2013-09-20

**Authors:** Yinong Chong, Steven M. Frenk

**Figure:**
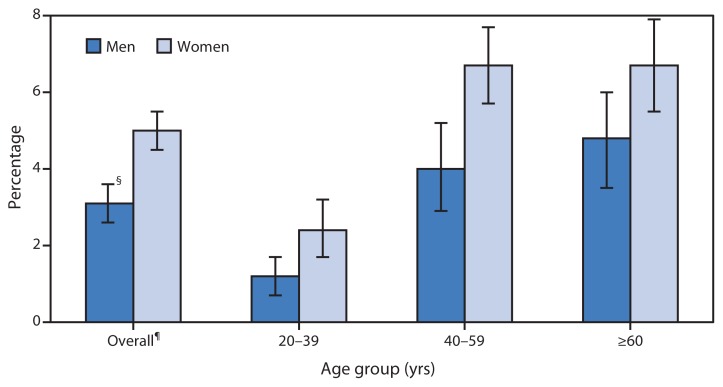
Percentage of Adults Aged ≥20 Years Who Used Prescription Sleep Aids* in the Past 30 Days,^†^ by Age Group and Sex — National Health and Nutrition Examination Survey, United States, 2005–2010 ^*^ Includes hypnotic drugs (e.g., butabarbital, chloral hydrate, estazolam, eszopiclone, flurazepam, quazepam, ramelteon, temazepam, triazolam, zaleplon, and zolpidem) and antidepressant drugs with sedative function (e.g., amitriptyline, doxepin, mirtazapine, and trazodone). ^†^ Based on response to the question, “Have you taken or used any medicines for which a doctor’s or dentist’s prescription is needed in the past month?” Respondents who answered affirmatively were asked to report the name, duration of use, and main reason for each product used. ^§^ 95% confidence interval. ^¶^ The overall estimate is age-adjusted to the 2000 projected U.S. standard population using the age groups 20–39, 40–59, and ≥60 years.

During 2005–2010, women were more likely to use a prescription sleep aid than men (5.0% versus 3.1%). Within the three age groups examined, women also were more likely to use a prescription sleep aid than men. For both men and women, adults aged 20–39 years reported lower use of sleep aids than adults aged 40–59 years and ≥60 years.

**Source:** Chong Y, Fryar CD, Gu Q. Prescription sleep aid use among adults: United States, 2005–2010. NCHS data brief no. 127. Hyattsville, MD: US Department of Health and Human Services, CDC; 2013. Available at http://www.cdc.gov/nchs/data/databriefs/db127.htm.

